# Functional status in ICU survivors and out of hospital outcomes

**DOI:** 10.1186/2197-425X-3-S1-A178

**Published:** 2015-10-01

**Authors:** JE Rydingsward, CM Horkan, KM Mogensen, FK Gibbons, K Amrein, KB Christopher

**Affiliations:** Department of Rehabilitation, Brigham and Women's Hospital, Boston, United States; Department of Medicine, Brigham and Women's Hospital, Boston, United States; Department of Nutrition, Brigham and Women's Hospital, Boston, United States; Pulmonary and Critical Care Medicine, Massachusetts General Hospital, Boston, United States; Division of Endocrinology and Metabolism, Department of Internal Medicine, Medical University of Graz, Graz, Austria; Renal Division, Brigham and Women's Hospital, Boston, United States

## Introduction

While studies suggest that functional status may be modifiable in the ICU, limited information exists in ICU survivors regarding the association between functional status at hospital discharge and adverse events following hospital discharge.

## Objectives

To examine the association between functional status at hospital discharge in survivors of critical care and risk of 90-day all-cause mortality after hospital discharge.

## Methods

We performed a retrospective cohort study in one Boston teaching hospital on 10,343 adults who received critical care between 1997 and 2011 and survived hospitalization. All patients had a formal evaluation by a physical therapist at hospital discharge. The exposure of interest was functional status determined by physical therapy evaluation. Patients were assessed on bed mobility (roll side to side, supine to sit, sit to supine), transfers (sit to stand, stand to sit, bed to chair), and gait (level ambulation, stairs). The primary outcome was 90-day post hospital discharge all-cause mortality obtained from the US Social Security Administration Death Master File which has high sensitivity and specificity for mortality. We used logistic regression to describe how 90-day post hospital discharge mortality differed with functional status at hospital discharge. We also evaluated how the odds of 30-day readmission differed with functional status at hospital discharge.

RESULTS

In an logistic regression model adjusted for gender and the Acute Organ Failure score, a validated ICU risk-prediction score derived from age, race, surgery vs. medical patient type, comorbidity, sepsis and acute organ failure covariates, the lowest quartile of functional status at hospital discharge was associated with a 6 fold increased odds of 90-day post-discharge mortality compared to patients with independent functional status [OR = 5.64 (95%CI 3.97-8.01; P < 0.001)]. The adjusted odds of 30-day readmission in patients with the lowest quartile of functional status at discharge was 1.3 fold higher than patients with independent functional status [OR 1.28 (95%CI 1.07-1.52; P = 0.008)]. In patients who had at least 7 days of physical therapy treatment prior to discharge (N = 2,293), the adjusted odds of 90-day post-discharge mortality in patients with marked improvement in functional status at discharge was 64% less than patients with no change in functional status after adjustment for the acute organ failure score, gender and the number of days between initial and discharge physical therapy assessment [OR 0.36 (95%CI 0.24-0.53); P < 0.001].

## Conclusions

Lower functional status at hospital discharge in survivors of critical illness is associated with increased post-discharge mortality and hospital readmission. Further, patients whose functional status improves during hospitalization have decreased odds of post-discharge mortality. ICU survivors with low functional status are a high risk group for adverse outcomes following hospital discharge.

